# Supplementary material for: Bayesian refinement of protein structures and ensembles against SAXS data using molecular dynamics
Source: PLoS Comput Biol. 2017 Oct 18;13(10):e1005800. doi: 10.1371/journal.pcbi.1005800 (PMC5662244; doi:10.1371/journal.pcbi.1005800)
Supplement: S1 Archive — (BZ2) [file pcbi.1005800.s011.tar.bz2 › gromacs_4.62_bayesian_saxs_refi/share/html/online/g_filter.html]

g\_filter


| g\_filter Main Table of Contents |

**VERSION 4.6  
Sat 19 Jan 2013**

---

### Description

g\_filter performs frequency filtering on a trajectory.
The filter shape is cos(π t/A) + 1 from -A to +A, where A is given
by the option -nf times the time step in the input trajectory.
This filter reduces fluctuations with period A by 85%, with period
2\*A by 50% and with period 3\*A by 17% for low-pass filtering.
Both a low-pass and high-pass filtered trajectory can be written.

Option -ol writes a low-pass filtered trajectory.
A frame is written every -nf input frames.
This ratio of filter length and output interval ensures a good
suppression of aliasing of high-frequency motion, which is useful for
making smooth movies. Also averages of properties which are linear
in the coordinates are preserved, since all input frames are weighted
equally in the output.
When all frames are needed, use the -all option.

Option -oh writes a high-pass filtered trajectory.
The high-pass filtered coordinates are added to the coordinates
from the structure file. When using high-pass filtering use -fit
or make sure you use a trajectory that has been fitted on
the coordinates in the structure file.

### Files

| option | filename | type | description |
| --- | --- | --- | --- |
| **-f** | traj.xtc | Input | Trajectory: xtc trr trj gro g96 pdb cpt |
| **-s** | topol.tpr | Input, Opt. | Structure+mass(db): tpr tpb tpa gro g96 pdb |
| **-n** | index.ndx | Input, Opt. | Index file |
| **-ol** | lowpass.xtc | Output, Opt. | Trajectory: xtc trr trj gro g96 pdb |
| **-oh** | highpass.xtc | Output, Opt. | Trajectory: xtc trr trj gro g96 pdb |

### Other options

| option | type | default | description |
| --- | --- | --- | --- |
| **-[no]h** | bool | no | Print help info and quit || **-[no]version** | bool | no | Print version info and quit || **-nice** | int | 19 | Set the nicelevel || **-b** | time | 0 | First frame (ps) to read from trajectory || **-e** | time | 0 | Last frame (ps) to read from trajectory || **-dt** | time | 0 | Only use frame when t MOD dt = first time (ps) || **-[no]w** | bool | no | View output .xvg, .xpm, .eps and .pdb files || **-nf** | int | 10 | Sets the filter length as well as the output interval for low-pass filtering || **-[no]all** | bool | no | Write all low-pass filtered frames || **-[no]nojump** | bool | yes | Remove jumps of atoms across the box || **-[no]fit** | bool | no | Fit all frames to a reference structure |

---

http://www.gromacs.org  
gromacs@gromacs.org
